# The dynamic mechanism of noisy signal decoding in gene regulation

**DOI:** 10.1038/srep42128

**Published:** 2017-02-08

**Authors:** Peijiang Liu, Haohua Wang, Lifang Huang, Tianshou Zhou

**Affiliations:** 1 School of Mathematics, Sun Yat-Sen University, Guangzhou 510275, People’s Republic of China; 2 Department of Mathematics College of Information Science and Technology Hainan University, Haikou 570228, People’s Republic of China; 3 School of Mathematics and Information Science, Guangzhou University, Guangzhou 510006, People’s Republic of China

## Abstract

Experimental evidence supports that signaling pathways can induce different dynamics of transcription factor (TF) activation, but how an input signal is encoded by such a dynamic, noisy TF and further decoded by downstream genes remains largely unclear. Here, using a system of stochastic transcription with signal regulation, we show that (1) keeping the intensity of the signal noise invariant but prolonging the signal duration can both enhance the mutual information (MI) and reduce the energetic cost (EC); (2) if the signal duration is fixed, the larger MI needs the larger EC, but if the signal period is fixed, there is an optimal time that the signal spends at one lower branch, such that MI reaches the maximum; (3) if both the period and the duration are simultaneously fixed, increasing the input noise can always enhance MI in the case of transcription regulation rather than in the case of degradation regulation. In addition, we find that the input noise can induce stochastic focusing in a regulation-dependent manner. These results reveal not only the dynamic mechanism of noisy signal decoding in gene regulation but also the essential role of external noise in controlling gene expression levels.

Cells are often exposed to complex environments and need to respond reliably to external signals for better survival. There are two main mechanisms to process (in fact encode) signals in cellular signaling pathways^1^,^2^,^3^,^4^,^5^,^6^: amplitude modulation (AM) in which the absolute concentration of an internal signaling molecule encodes the input signal, and frequency modulation (FM) in which the period between successive bursts represents the stimulating signal. Although these two signal-encoding mechanisms have been observed in signaling pathways, the question of how a dynamic and noisy signal is finally decoded into the genetic information still remains highly elusive.

There is experimental evidence to support that AM or FM or both can occur in gene regulation. Here we list several related examples. First, p53, a tumor transcription factor (TF), may respond to the UV radiation in an AM or a FM manner^1^,^2^,^3^. Second, in the budding yeast *Saccharomyces cerevisiae*, the nuclear concentration of the TF Msn2 is proportional to the H_2_O_2_ concentration under oxidative stress, implying an AM mechanism^7^. Third, the TF Crz1 that responds to a calcium stimulus regulates at least a hundred target genes after entering the cellular nucleus in unsynchronized bursts^8^. The level of stimulus impacts only the burst frequency but not the burst amplitude and the duration, suggesting an FM mechanism. Fourth, Albeck, *et al*.^9^, analyzed two critical aspects of the EGF-stimulated ERK/MAPK pathway (a key conduit for cellular proliferation signals and also a therapeutic target in many cancers): the mechanism by which signal strength is encoded and the response curve relating signal output to proliferation, and found that under steady-state conditions, ERK is activated in discrete, asynchronous pulses with frequency and duration determined by extracellular concentrations of EGF spanning the physiological range. In addition, the bursty FM is also found in bacteria and mammals, e.g., during energy-depletion stress, the bacterium *Bacillus subtilis* regulates about 150 downstream genes by activating a certain TF in discrete stochastic pulses^10^; isoform NFAT4 in activated T-cells shows similar behavior^11^. These indicate that the bursty FM is a general modulation scheme across different cell types.

There have been theoretical works^4^,^6^,^12^,^13^,^14^,^15^ to explore the mechanisms of amplitude and frequency decoding as well as the reliability of these two decoding schemes. For example, Tostevin, *et al*.^6^, studied the dynamics of gene promoter in a simple model in response to oscillating or constant TF signals, and found that in biologically relevant regimes of system parameters, a deterministic and oscillating input signal can produce a more constant protein level than a constant input signal. There have been other theoretical works^1^,^4^,^5^,^13^ to investigate advantages and disadvantages of AM and FM. For example, Micali *et al*.^4^, analyzed another simple model for a single receptor that can either signal continuously whenever a ligand is bound, or produce a burst in signaling molecule upon receptor binding, and showed that FM is more accurate than AM merely for a single receptor with fast signaling, whereas the latter is more accurate than the former only in slow gene regulation and with signaling by multiple receptors. Although these works or others^3^,^5^,^7^,^10^,^14^,^15^ well revealed the mechanisms of how signals are decoded either in signaling networks or in gene regulation, the used models considered only the case that input signals are deterministic.

In biological systems, however, external signals and further TFs activated by these signals are in general dynamic and noisy. In fact, signaling pathways can induce different dynamics of TF activation. For example, Hao *et al*.^5^, showed that the budding yeast general stress–responsive TF Msn2 acts as a tunable signal processor that might track, filter, or integrate signals in an input-dependent fashion. At the same time, they additionally pointed out that this tunable signal processing appears to originate from dual regulation of both nuclear import and export by phosphorylation, and emphasized that versatile signal processing by Msn2 is important for generating distinct dynamic responses to different natural stresses. More recently, Hansen and O’Shea^1^ applied information theory to quantify how much gene expression information the yeast TF Msn2 can transduce to target genes in the amplitude or frequency of its activation dynamics, and found that although the amount of information transmitted by Msn2 to single target genes is limited, information transduction can be increased by modulating promoter cis-elements or by integrating information from multiple genes.

Motivated mainly by these two works, we introduce a biologically reasonable model of stochastic transcription, where the transcription rate or the mRNA degradation rate is supposed to be regulated directly by a TF signal. Different from previous studies, however, our model considers that this regulatory signal (input) may not only be dynamic and noisy but also regulate the expression level (output) of the target gene in different manners. Apart from considering two common kinds of signals: amplitude signal, i.e., the amplitude of a signal is changeable but its frequency is fixed, and frequency signal, i.e., the frequency of a signal is changeable but the amplitude is invariant, we also consider the duration of a noisy signal, where the duration is defined as the time length from a pulse to the next pulse of the noisy signal (hence it describes the degree of fluctuations in the signal noise’s frequency).

Another motivation is due to consideration of stochastic focusing (SF), an important kind of biological phenomenon and also a representative mechanism by which signals are enlarged. In previous studies on SF^16^,^17^,^18^,^19^,^20^, a fluctuated signal was supposed as an amplitude-modulated one and the basic requirement for SF is that the signal is rapidly fluctuated and the response function of the output signal to the input signal is nonlinear. For instance, as demonstrated in a basic enzymatic reaction scheme^16^, the basic (empirically derived) conditions for SF are that the magnitude of active enzyme fluctuations is significantly large compared to the mean number of active enzymes, while the total number of enzymes may be very low. Moreover, the only regulatory way is that the input noisy signal acts directly on a decay rate. In addition, we note that a frequency-modulated signal was not taken into consideration in the existing references. In this article, we will address the question of whether an amplitude-modulated or a frequency-modulated input signal induces the SF of the output.

Our investigation focuses on effects of the noise in the upstream signal on the downstream mRNA expression level, with a main result that the input noise can induce the SF of the output (i.e., mRNA). More importantly, to reveal the decoding mechanism of the dynamic and noisy signal, similar to ref. 1, we use the mutual information (MI) between the input and the output to quantify the information loss but use energy consumption (EC) quantified by the entropy production rate of the mRNA to quantify the cost of signal transduction (or information transmission). By model analysis, we find some interesting phenomena that have not been observed in cases of deterministic inputs. Importantly, we elucidate the dynamic mechanism of how an upstream dynamic and noisy signal is decoded by a downstream gene.

## Method

### Model description

**Figure 1:**
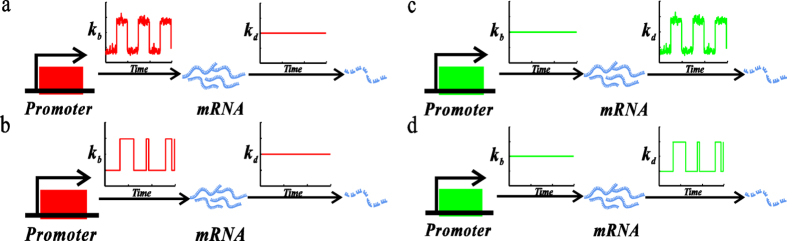
Schematic description for a model of stochastic transcription with signal regulation: (**a**) the mRNA decay rate, *k*_*d*_, is constant but the transcription rate, *k*_*b*_, is regulated by an input signal, the amplitude of which is fluctuated but the period is fixed; (**b**) the decay rate is constant but the transcription rate is regulated by an input signal, the period of which is fluctuated but the amplitude is invariant; (**c**) the transcription rate is constant but the decay rate is regulated by an input signal, the amplitude of which is fluctuated but the period is fixed; (**d**) the transcription rate is unchanged but the decay rate is regulated by an input signal with fluctuated period and invariant amplitude.

As pointed out in the introduction, a TF signal may not only be dynamic and noisy but also regulate the expressions of target genes in different manners. For analysis convenience, we consider a common model of stochastic transcription, where the gene is supposed to be directly transcribed into mRNA. Furthermore, we assume that a dynamic and noisy TF regulates directly either the transcription rate or the mRNA degradation rate, i.e., we assume that the change in each of these two rates represents dynamics of the TF activation or the input signal. In addition, we assume that the TF signal occurs in a pulsating manner (in fact, oscillating signals exist widely in gene regulatory systems^21^,^22^). Alternatively, we assume that a common ON-OFF model generates a dynamic, fluctuated signal, which regulates, as a TF, the expression of the downstream gene^13^,^23^,^24^. However, we distinguish a dynamic and noisy TF as an amplitude-fluctuated signal or a frequency-fluctuated signal. Thus, there are four possibilities in total^6^, referring to Fig. 1. For convenience, we call a TF as transcriptional signal if it regulates the transcription rate, and the corresponding model as a transcription-regulated model, whereas a TF as degradation signal if it regulates the mRNA degradation rate, and the corresponding model as a degradation- regulated model.

According to the above assumptions, the gene regulatory model shown in Fig. 1 can essentailly be viewed as a birth-death process but the current mRNA production (i.e., transcription) or degradation rate may be time-dependent. Thus, if we denote by DNA the gene, by *K*_*b*_(*t*) the temporal transcriptional rate (unit: *μM*/sec), and by *K*_*d*_(*t*) the temporal degradation rate (unit: *μM*/sec), then the gene model under consideration can be described by the following two biochemical reactions


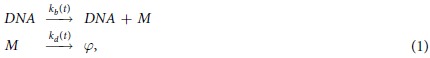


where 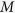 stands for mRNA and ϕ for degradation. Furthermore, if *k*_*b*_(*t*) or *k*_*d*_(*t*) is allowed to be stochastically fluctuated (either in the amplitude or in the frequency of the input signal), then equation (1) can represent all the four possible cases in Fig. 1. This will bring us analysis convenience.

Next, we establish our chemical master equation to be studied. Let *P(m, t*) represent the probability that mRNA has *m* copy numbers at time *t*. Then, the master equation takes the form


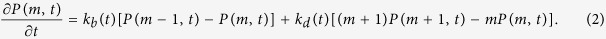


Noting that *P(m, t*) should be understood as a probability conditional to the input signal in the case that the signal is dynamic and noisy. In particular, equation (2) corresponds to the transcription-regulated model if *k*_*d*_(*t*) is constant whereas to the degradation-regulated model if *k*_*b*_(*t*) is constant. One will see that if *k*_*b*_(*t*) and *k*_*d*_(*t*) are all determinsitic, then equation (2) can be analytically solved (see contents in the following paragraphs). If one of these two rates is dynamic and noisy, however, then analytically solving equation (2) seems very difficult. In this case, we will develop a numerical method, which is actually a modified version of the famous Gillespie algorithm (also see contents in the following paragraphs or Appendix A).

Since the input signal is assumed to be pulsating or oscillating, we introduce the so-called “ON-state”, “OFF-state”, “ON-Time” and “OFF-Time” of the signal for convenience. In the case that transcription rate is regulated, an ON-state corresponds to an upper branch of the oscillating signal whereas an OFF-state to a lower branch (referring to Fig. 1a and b). For the transcription-regulated model, the ON-time is defined as the time that the signal spends in the higher state whereas the OFF-time as the time that the signal spends in the lower state. For the degradation-regulated model, however, the definitions of ON-time and OFF-time should be just opposite to those for the transcription-regulated model since for the former, the larger the decay rate is, the lower the mean value of mRNA is. Note that the ON-time and the OFF-time are all random variables in the noisy FM case but are deterministic in the AM case. In general, these times and their means can be obtained only by numerical calculation. We point out that the above definitions in the case that the transcription rate is regulated may be analogous to those in the case of the common ON-OFF model^25^. In addition, for an AM signal, the ON-time and the OFF-time are all constants, whereas a FM signal does not fluctuate in its amplitude but in the ON-time and the OFF-time. In a word, all the terminologies should be understood in natural manners.

### A modified version of the Gillespie algorithm

The standard Gillespie algorithm^26^ has been extensively used to simulate chemical systems where the reaction propensities are time-independent. Since the transcription rate or degradation rate is currently dynamic and noisy in our case, implying that the process described by equation (2) is non-Markovian, this algorithm cannot be directly applied but needs to be modified. Here we propose a modified version of the standard Gillespie algorithm to solve equation (2).

Note that for an oscillating input signal, the noise emerges mainly in two ways: fluctuations in the amplitude and those in the timing of signal pulses. First, we consider the amplitude-fluctuated case. On each occasion, the oscillating signal switches on the amplitude *k*_*b*_(*t*). Similar to ref. [6], we sample *k*_*b*_ from a log-norm distribution (we point out that if a different distribution, e.g., a Gamma distribution, is used, then all the qualitative results to be obtained are kept invariant) with two given mean values 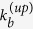 and 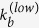, which represent the upper and lower bound of the pulsating signal without noise respectively, referring to Fig. 1b and d. Note that in this case, the mean width of the noisy signal is set as a constant. Then, we consider the frequency-fluctuated case. In this case, fluctuations in the amplitude are set as zero but the widths of the signal in the upper and lower parts are generated also from a log-norm distribution. As such, we can calculate ON-time and OFF-time according to their definitions. In calculation, we keep the variance of the frequency signal at the same value. Completely similarly, we can give a numerical algorithm for calculatig *k*_*d*_(*t*).

Once *k*_*b*_(*t*) and *k*_*d*_(*t*) are numrically determined using the above numerical schemes, the left calculation steps are the same as those in the standard Gillespie algorithm. See Appendix A for more details.

### Mutual information and energetic cost

As early as 1948, Shannon found that information theory can well quantify information transduction across a singaling pathway (or a channel) between an input signal and an output signal^1^,^12^,^27^. If a signaling pathway is noisy, a known signal input will result in a distribution of the signal output. This represents a loss of information since the signal input can no longer reliably be learned from observing the signal output. Mutual information (MI), MI(*X, Y*), which quantifies the amount of information about the signal input (*X*) that can be obtained by observing the signal output (*Y*), is mathematically defined as


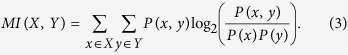


where *P(X, Y*) is the joint probability distribution of random variables *X* and *Y, P(X*) and *P(Y*) are the corresponding marginal distributions, respectively. Since the MI is usualy measured in bits, we use 2 as the base of logarithm^1^,^27^. In this paper, the MI defined in equation (3) is used to quantify information transmission. See the Appendix B for more details.

If a signal input can be precisely controlled and the signal output distribution can be precisely measured, then the above information theory can be applied. In our case, the time-dependent and fluctuated birth or death rate, *k*_*b*_(*t*) or *k*_*d*_(*t*), is taken as a signal input whereas the downstream mRNA, *m*, as the signal output. In numerical calculation, we utilize the above-proposed schemes to generate the time-series data for the transcription and degradation rates, and the above modified Gillespie algorithm to obtain the time-series data for the mRNA level. Thus, we can calculate the joint probability distribution of the input and output signals, as well as the respective marginal distributions.

From the viewpoint of thermodynamics, signal transduction is a non-equilibrium process since an input signal is, e.g., pulse or irreversibility which can break detailed balance. From the perspective of information theory, the entropy production rate is precisely the amount of energetic consumption (EC)^28^,^29^. For a detailed-balance system, there is no EC, while for a non-equilibrium steady-state system, there is EC^30^. Therefore, a signal transduction process necessarily consumes energy. EC can reflect the cost of information decoding, but how energy is consumed in an information- decoding process is unclear.

Mathematically, EC is calculated according to the following formula^22^,^31^,^32^.


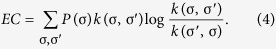


Here, *k(σ, σ*′) stands for the transition probability from state *σ* to state *σ*′, and *P(σ*) represents the probability that the underlying system is in state 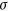. In our case, for a given set of time-series data for *k*_*b*_(*t*) or *k*_*d*_(*t*), if the time-dependent mRNA distribution, *P(m, t*), is given by the modified Gillespie algorithm, then equation (4) will become


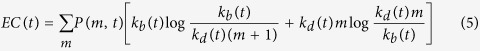


See Appendix C for its derivation.

## Result

### Analytical distribution

According to formula (5), we know that to calculate EC, the key is to derive the mRNA distribution *P(m, t*). For this purpose, i.e., in order to solve equation (2) analytically, we assume that *k*_*b*_(*t*) and *k*_*d*_(*t*) are two known time-dependent functions. In this case, if we introduce the probability-generating function 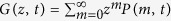 for *P(m, t*), then equation (2) can be transformed into the following partial differential equation


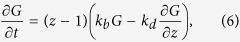


For equation (6), we seek for the solution of a particular form 
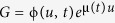
 with *u* = (*z* − 1), where ϕ(*u, t*) and *μ(t*) are two functions to be determined. Substituting this setting into equation (6) and eliminating the factor 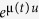 will lead to the following partial differentil equation


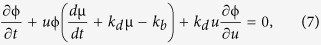


Motivated by studying a birth-death process with constant rates, we choose *μ(t*) such that it satisfies the following ordinary differential equation


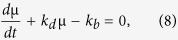


Such a choice of *μ(t*) is reasonable since if two rates *k*_*b*_ and *k*_*d*_ are constants, the constant ratio *μ* = *k*_*b*_/*k*_*d*_ is reduced to the known case. With this special choice of *μ(t*), equation (7) can be simplified as


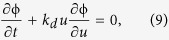


Thus, by applying the characteristic line method, we can easily get the general solution of equation (9), expressed by


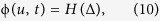


where *H* is an arbitrary function of 
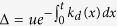
, and will be later specified if initial conditions are considered. Note that the solution to equation (8) is given by


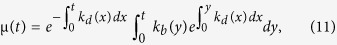


since equation (8) is a linear differential equation in *μ*.

Without loss of generality, we assume that the initial mRNA has *m*_0_ molecules. Then, the function *G(z*, 0) is given by


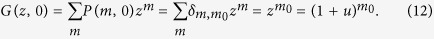


Owing to 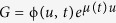 with *μ*(0) = 0, we know


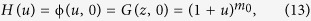


which thus determines the function *H*. It is easily to verify that the function


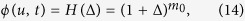


is the solution of equation (9) satisfying the initial condition (13). Furthermore, if the initial number of mRNA is zero, i.e., *m*_0_ = 0, then the solution to equation (6) with time-dependent rates *k*_*b*_(*t*) and *k*_*d*_(*t*) can be expressed as


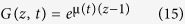


Thus, according to the relationship between the probaiblity density function and the generating function, we can obtain the time-dependent mRNA distribution, that is,


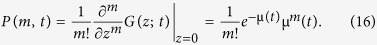


This indicates that the time-dependent ratio μ(*t*) is only a factor impacting the mean value of mRNA and the shape of the corresponding probability distribution. Moreover, if the function μ(*t*) is a constant, then the distribution given by equation (16) reproduces the known result for the birth-death process with constant rates.

If either the transcription rate *k*_*b*_ or the mRNA decay rate *k*_*d*_ is a random variable, implying that *μ(t*) follows some distribution, *Q(t*), then according to the distribution property^33^, we know that the resutling distribution *R(m, t*) should be the convolution of *Q(t*) and *P(m, t*), that is,


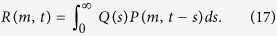


After having derived the analytical mRNA distribution, we next perform numerical simulation. In the demonstration of our numerical results, we will use the letter ‘D’ to represent the duration of an input noisy signal, the symbol ‘Var’ to represent the variance of this signal in the case that the dynamic transcriptional rate is sampled from a log-norm distribution, and the symbol ‘Noise’ to represent the ratio of the variance to the square of the mean value of the FM noisy signal, i.e., to represent the input noise.

### Increasing the input noise facilitates information transmission but is at the cost of EC: the AM case

In this subsection, we focus on the case of the AM signal (Fig. 1a,c). Note that in this case, the input signal fluctuates only in the amplitude but not in the period length, and the transcription (or decay) rate is modulated whereas the decay (or transcription) rate is kept unchanged.

### The effects of ON- and OFF-times on MI

**Figure 2:**
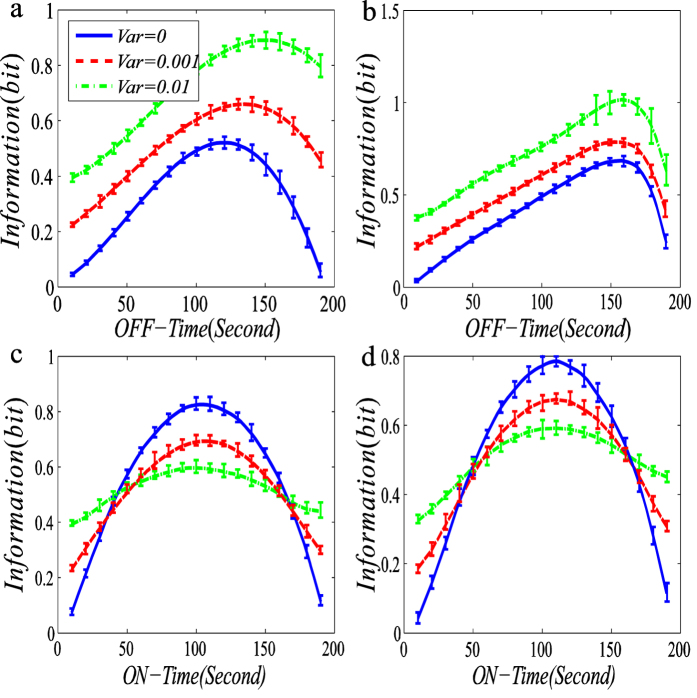
Dependences of mutual information (unit: bit) on ON-time and OFF-time (unit: second) for three fixed variances of input noisy signals are shown with error bars: the case of amplitude-modulated signal. The indicated “information” represents the mutual information between the input and the output, which is calculated according to equation (3) in the main text. The blue line represents the determinstic signal (without noise); the red dash line and the dot dash green line stand for the variance sizes of the signals being 0.001 and 0.01 (unit: *μM*), respectively. (**a**,**b**) The transcription rate is regulated by an oscillating noisy signal while the decay rate is kept constant, where in (**a**,**b**), the mean values of the upper and the lower amplitudes of the signal are set as 0.8 and 0.1 (unit: *μM*) respectively, and in (**b**), the decay rate is set at 0.05 (unit: *μM*/sec) and the transcriptional signal is the same as in (**a**). (**c**,**d**) The decay rate is regulated by an oscillating noisy signal while the transcription rate is kept constant. In the first column, the mean value of the output mRNA is fixed at 15 (unit: *μM*) whereas in the second column, the mean value is not fixed, and in (**c**), the mean values of the upper and lower signals are set as 0.1 and 0.025 respectively, whereas in (**d**), the decay signal is the same as in (**c**), the transcription rate is set at 0.5 (unit: *μM*/sec). In all cases, the sigal duration is fixed at 3 (unit: second), the time length of the whole period is set at 200 (unit: second), and each curve represnts the average over 1000 realizations.

Here, we plot the dependences of MI in equation (3) on ON-time and OFF-time respectivley, referring to Fig. 2.

From this figure, we first observe that the MI function of ON-Time or OFF-Time between the input signal and the output signal is upward convex in the case of no noise. However, with increasing the variance of the input noisy signal, this convex function becomes flatter. Second, we observe that in the case that the transcription rate is regulated, the MI increases with the increase in the variance of the input signal for a fixed OFF-time, referring to Fig. 2a and b, while in the case that the degradation rate is regulated, the change tendency of MI is basically opposite to that in the former case, referring to Fig. 2c and d. Specifically, the size of the MI decreases with the increase in the variance of the input signal for a fixed moderate ON-time. Third, in all the four cases, there is an optimal ON-time or OFF-time such that the MI function reaches a maximum, but this maximum will gradually become unapparent as the noise variance of the input signal increases. Fourth, the maximum may drift with the increase in the variance of the input noisy signal in the case that the transcription rate is regulated. Finally, the errors near the maximum are in general larger than those in other places, implying that the former is more sensitive to the ON-Time than the latter.

It is a known fact that the larger the value of mutual information is, the better is the effect of information transmission. Therefore, the results shown in Fig. 2 imply that the noise in the input signal (or external noise) facilitates information transmission. This is one of the main results of this paper.

### The effects of ON- and OFF-times on EC

**Figure 3:**
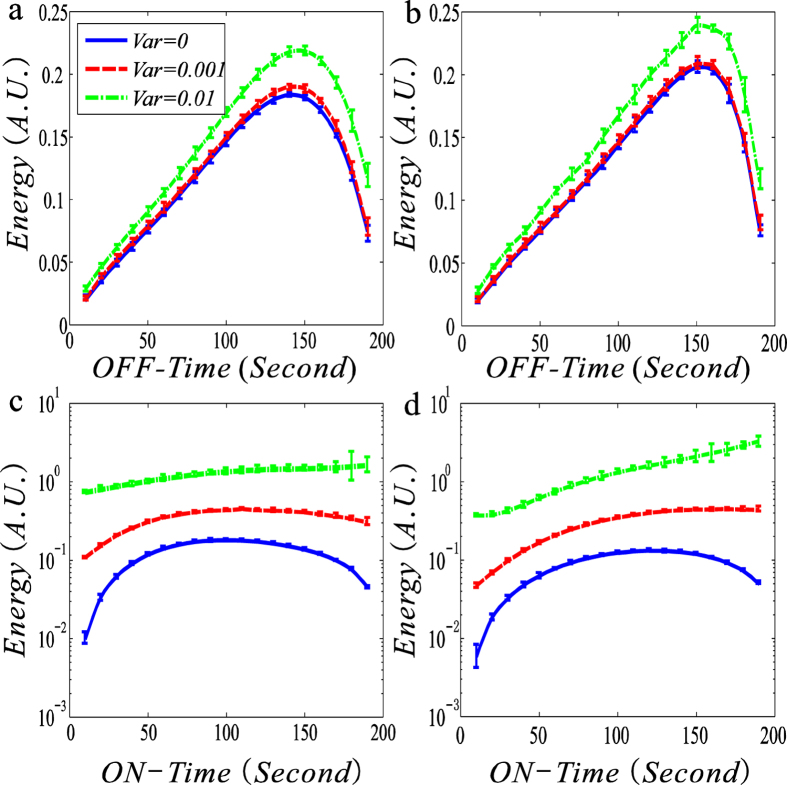
Dependences of energy consumption (no unit) on ON-time and OFF-time for three fixed variances of input noisy signals are shown with error bars: the case of amplitude-modulated signal. All settings and all the considered cases are the same as in Fig. 2.

Here, we check characteristics of EC in the information transmission process. For this, we plot the dependences of the EC calculated by equation (5) on ON-Time and OFF-Time respectivley, referring to Fig. 3.

From Fig. 3, we observe that the change tendency of EC is basically the same as that of MI in the case that the transcription rate is regulated (comparing Fig. 3a and b with Fig. 2a and b). For example, the EC function of OFF-time is upward convex, and there is an optimal ON-time or OFF-time such that this function arrives at a maximum. However, there are apparent differences in change tendency between Figs 2 and 3 in the case that the degradation rate is regulated (comparing Fig. 3c and d with Fig. 2c and d). For example, the EC always becomes larger for an arbitrarily fixed ON-time as the variance of the input noisy signal increases, and the EC function of OFF-time is not convex at all but finally becomes monotonically increasing after the variance is sufficiently large. Figure 3 indicates the fact that the larger the input noise is, the greater is the energy consumed.

**Figure 4:**
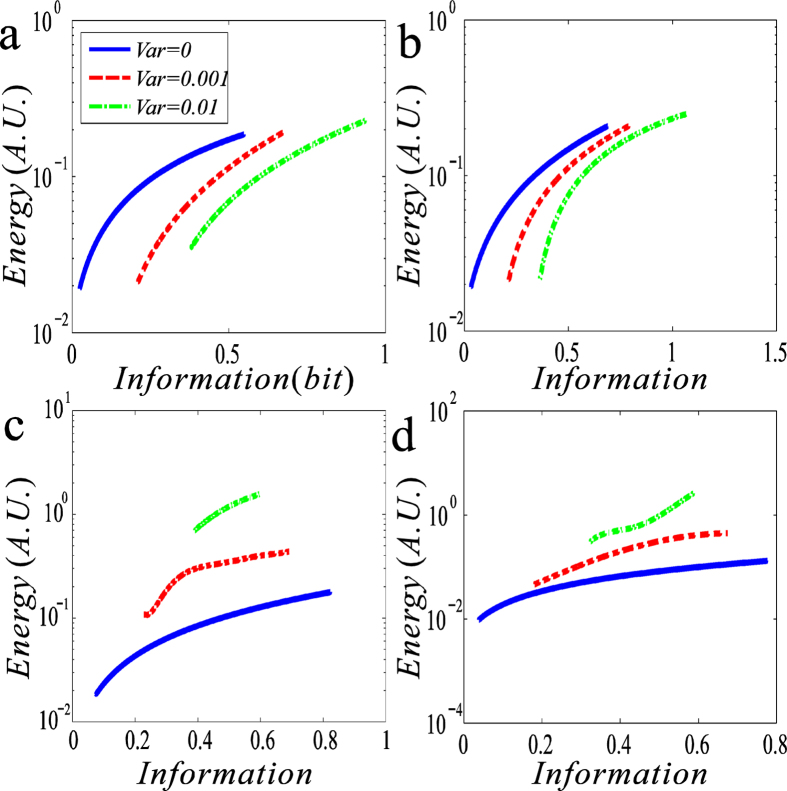
The relationship between energetic consumption (‘Energy’) and mutual inforation (‘Information’), where all settings and all the considered cases are the same as inFig. 2. It is shown that there is a positive correlation between ‘Energy’ and ‘Information’.

In summary, comparing Fig. 3 with Fig. 2, we first find in the case that the trancriptional rate is regulated, there is a positive correlation between the input-output MI and the EC, referring to Fig. 4. That is, the more the information is transmitted, the larger is the energy consumed, which is in accordance with our intuition. From Fig. 4, we observe that with increasing the variace in the input signal, this positive correlation becomes more apparent, implying that the larger input noise is more beneficial to the information transmission but this dissipates the more energy. Then, we find that in the case that the transcriptional rate rather than the degradation rate is regulated, the MI or the EC can arrive at a maximum value, and the maximum of the former appearing at the time as the length of OFF-time is about twice that of ON-time.

Here, we give intuitive interepretations for numerical results shown in Figs 2 and 3. For clarity, we consider only the case that the transcription rate is regulated. According to the addition rule of noise^34^, we know that the noise in output signal (i.e., mRNA) is equal to the noise in input signal (i.e., the external noise) plus the noise generated from the random birth-death of mRNA (i.e, the internal noise). Simply speaking, the input noise increases the output noise. However, the noise can be viewed as a kind of stochastic force, and the larger this force is, the more the energy is necessarily dissipated. Thus, an AM or a FM signal increases EC. In addition, the stronger the input noise is, the larger is the output noise, implying that the output and input signals are more closely correlated. Therefore, the result that the MI between the output and input signal becomes larger is not strange. For other regulation cases, similar intuitive explanations can also be given.

In addition, we give biological interpretations for numerical results shown in Figs 2 and 3. In biology, the DNA wrapping around nucleosomes is a stochastic process governed by diffusion^35^, e.g., the binding of chromatin remodeling factors to the promoter DNA. It is believed that promoter DNA wrapped around nucleosomes is very stable and has a typical lifetime that is longer than the timescale of transcription^36^. Note that asymmetrical mean times that a gene spends at ON and OFF states are a common phenomenon in gene expression^37^. In our case (Fig. 2a and b), we have shown that an asymmetrical ON-OFF gene motif has advantages in signal processing since it can transfer more information on the external signal to the internal gene expression. Thus, we conjecture that a cell would take advantage of such a simple network structure to copy with its uncertain external environment. For such a conjecture, Micali *et al*.^4^, ever gave a reasonable explanation, based on the fact that a receptor/ion channel can only detect information on the extracellular environment during unbound (OFF) time intervals (since an extracellular stimulus only affects the binding rate). Thus, the result shown in Fig. 2a and b, i.e., the amount of information transmission can be enlarged as the OFF-time is prolonged, is biologically resonable.

In the case that the decay rate is regulated (Fig. 1c), however, the maximum value of MI emerges at the time when the ON-time and OFF-time almost have the same value, implying that the maximum MI is drifted in this case. In spite of this, the enhancement of information transmission exhibits inconsistent tendency, where by ‘inconsistent’ we mean that the size of MI is not always lager under regulation of a big input noise signal than under regulation of a small input noise signal. For moderate values of OFF-time, the size of MI with small noise is much larger than that with big noise (Fig. 2c and d). Here, we give some (biological) interpretations. If an AM signal regulates the degradation rate, then the bigger noise should be able to enhance the information transimission since ON-time and OFF-time are asymmetric in a real case (in fact, for most genes, the former is singificantly larger than the latter). Even if OFF-time is much larger than ON-time, then the bigger noise can still result from the enhancement of information transmission. These indicate advanatges of AM signal in enhancing information tarnsimission if it regulates the transcription rate. In contrast, the noise strength of the external signal has a different influence on EC, e.g., the bigger the noise is, the more is the energy expended, independent of ON-time or OFF-time.

### Increasing the input noise facilitates information transmission but is at the cost of EC: the FM case

**Figure 5:**
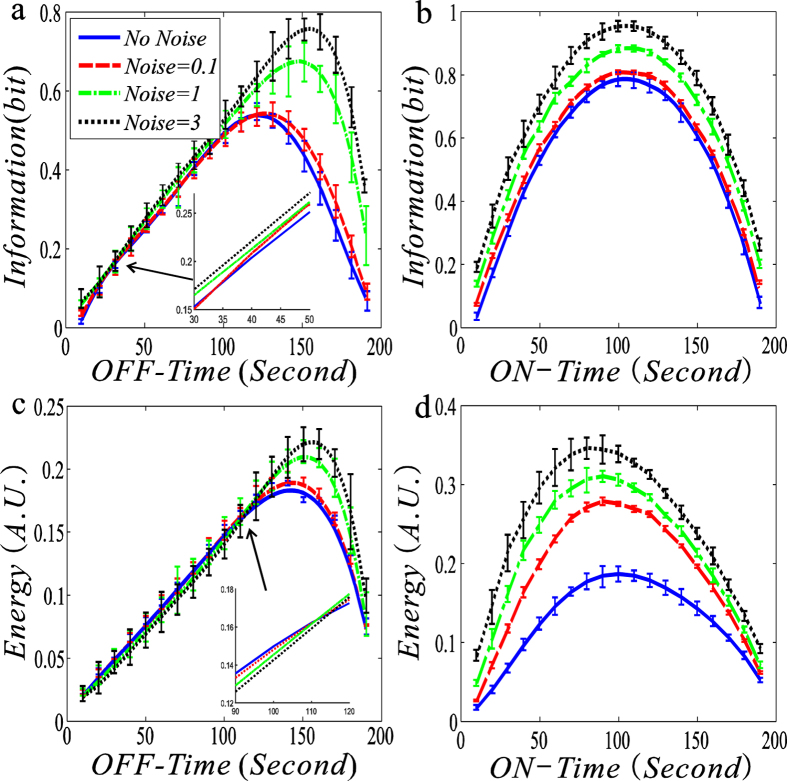
Dependences of mutual information/energy consumption on ON-time and OFF-time for four fixed variances of noisy input signal are shown with error bars: the case of frequency-modulated signal, where insets are locally diagrams corresponding to the parts indicated by arrows. The solid blue line, red dash line, dot-dash green line and dot black line correspond respectively to 0, 0.1, 1 and 3 input noise intensities. (**a**,**c**) The case that the transcription rate is regulated, where the upper and lower bounds of an oscillating signal are set as 0.8 and 0.1, respectively. (**b**,**d**) The case that the degradation rate is regulated, where the upper and lower bounds of an oscillating signal are set as 0.1 and 0.025, respectively. In (**a**,**b**), the mean mRNA level (output) is fixed at 15, and the changing ranges of ON-time and OFF-time are set from 10 to 190. The time length of a whole period is set at 200. Each curve represents the average over 1000 realizations.

The investigation here is basically similar to that in the previous subsection, but considers FM signal regulation. We use the relative fluctuation (i.e., ‘Noise’, which is defined as the ratio of the variance over the square of the mean) instead of the absolute fluctuation (‘Var’). A reason for this change is that the ‘Noise’ can better describe the randomness than the ‘Variance’ in the FM case. By numerical analysis, we obtain qualitative conclusions fundamentally similar to those obtained in the AM signal case. The specific numerical results are shown in Fig. 5.

From Fig. 5, we first observe that there is an optimal ON-time or OFF-time such that both the MI function and the EC function reaches a respective maximum value. Then, for a fixed ON-time or OFF-time, the size of the MI or the EC is fundamentally higher in the case of high noise than in the case of low noise, implying that increasing the noise in the input signal can facilitate the information transmission but is at the cost of EC. In addition, we observe that in the case that the OFF-time is below some value, the MI and the EC are insensitive to the input noise, referring to Fig. 5a and c. In this case, we plot two locally enlarged diagrams to help understand the differences in effects of input noise on MI, referring to the insets of Fig. 5a and c.

Comparing Fig. 5 with Fig. 2, we find that basically, the MI changes modestly with increasing the noise in the input signal in the FM case, implying that MI is insensitive to external noisy signals. Here by “basically” we mean that one case is exceptional, that is, if the noise of FM signal is large enough, the amplification effect of information is still apparent. We also see from Fig. 5 that the solid blue line and the dash red line correspond to almost the same amount of information transmission even if the noise strength is set as 0.1 or 0. More generally, if the noise in the input FM signal is weak, then the amplification effect of information will not be apparent (data are not shown). One more important point is that increasing the noise intensity of the FM signal will increase the amount of information transmission (Fig. 5) but intersection between curves will not appear as in Fig. 2c and d. We find from Fig. 5a that the amount of information transmission is almost in the same value but the EC gets a slightly lower value when the ON-time is much larger than the OFF-time. Once the value of OFF-time exceeds about 120 (Fig. 5a), the functional role of noise will become apparent. This is in accordance with the general consensus of what a gene has asymmetrical dwell times in ON-state and OFF-state is better for information transmission. As the noise intensity increases, the maximum MI will appear at a smaller ON-time in the transcription-regulated model but this phenomenon does not take place in the degradation-regulated model.

Another interesting result is that in the frequency-modulted model, more information can be transferred in the case that the deacay rate is regulated than in the case that the transcription rate is regulated. Comparing Fig. 5b with Fig. 2c, we find that in the former case, the downstream gene can obain more information from the upstream signal at the less cost of energy (Figs 3c and 5d) in the case of FM signal than in the case of AM signal. On contrary, the amplitude-modulated signal transfers more information than the frequency-modulated signal when the transcriptional rate is regulated by an external stimuli (Figs 2a and 5a) but the EC is almost kept at the same level. Therefore, we reckon that in information transmission with EC, an amplitude-modulated signal would be better than a frequency-modulated signal for the transcription-regulated model, whereas the latter would be superior to the former for the degradation-regulated model. This conjecture would be significant from the viewpoint of synthetic biology.

### Increasing the signal duration can amplify MI and reduce EC simultanously

**Figure 6:**
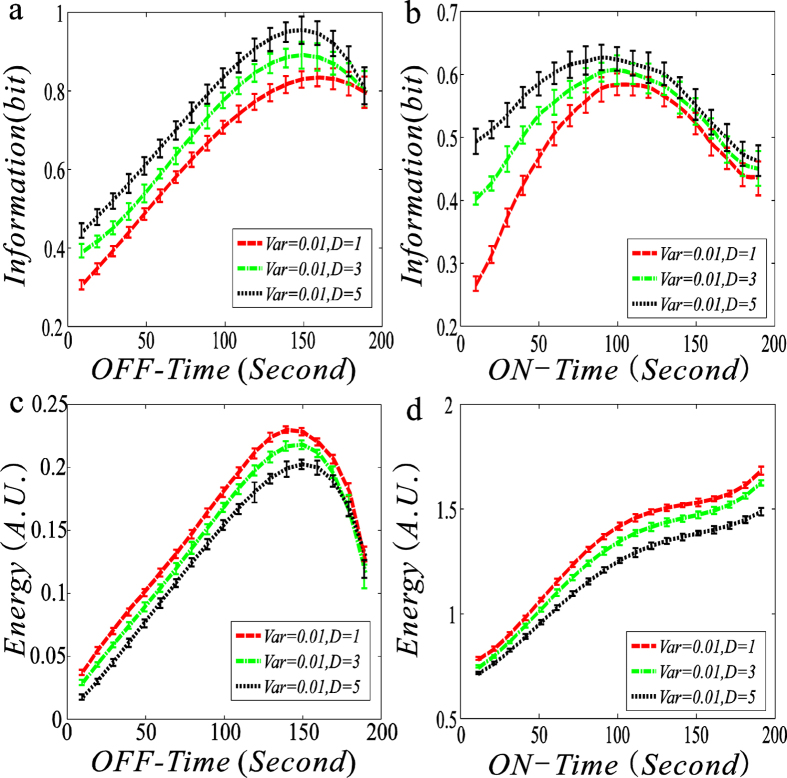
Dependences of mutual information/energy consumption on ON-time and OFF-time for fixed durations of input signal are shown with error bars. The left column (**a**) and (**c**) corresponds to the case that the transcription rate is reglated whereas the right column (**b**,**d**) to that the case that the degradation rate is reglated. In (**a**,**c**), the mean values for the up and down branches of the input pulse signal are set as 0.8 and 0.1 respectively, whereas in (**b**,**d**), the mean values for the up and down branches as 0.1 and 0.025, respectively. In all the four diagrams, ‘D’ represents the duration of the input signal, the mean value of the output is fixed at 15, and each curve represents the average over 1000 realizations.

Duration is a characteristic of many sginals. In previous works^8^,^10^, the effect of this factor on information transmission was neglected. Here, we investigate how the signal duration impacts information transmission with EC. For clarity, we separately consider two regulation cases: the transcription rate and the mRNA degradation rate are regulated by a dynamic and noisy signal. In addition, we plot the dependences of the MI and the EC on ON-time/OFF-time in three cases of signal duration with a fixed variance of the input noisy signal. The numerical results are shown in Fig. 6.

We observe from Fig. 6 that the MI and EC functions as ON-Time or OFF-Time are fundamentally upward convex in both regulation cases, except for one situation where the EC function of ON-time is approximately monotonically increasing, referring to Fig. 6d. Moreover, in the other three situations, there is an optimal ON-time or OFF-time such that each of the MI function and the EC function reaches a maximum value. In addition, for a fixed ON-Time or OFF-Time, increasing the signal duration will increase the amount of MI, referring to Fig. 6a and b, but will decrease the size of EC, referring to Fig. 6c and d. We point out that if the variance of input signal is further increased (e.g., if it reaches 0.1), then the signal duration does not influence the size of MI or EC, indicating that the influence of signal duration on MI or EC is independt of signal variance.

Figure 6 implies that decreasing the fluctuation frequency of the upstream signal can enhance the decoding capacity of the downstream signal but reduce the EC at the same time. On the other hand, according to the definition, we know that the increase in the signal duration means that the time interval between two pulses of the signal becomes longer. Thus, the fact that the MI is amplified and the EC is reduced by increasing the signal duration implies that a longer signal duration has advantges in both amplifying MI and reducing EC than a shorter one. This fact was also validated by Raser when he studied eukaryotic gene expression^37^.

From Fig. 6, we also see that if the mRNA output is kept at the same level, then the transcription-modulated model can transfer more information but consume less energy than the degradation-modulated model, implying that the former is better than the latter under the same condition, in accordance with the previously-obtained results.

### Input noisy signal can induce stochastic focusing

**Figure 7:**
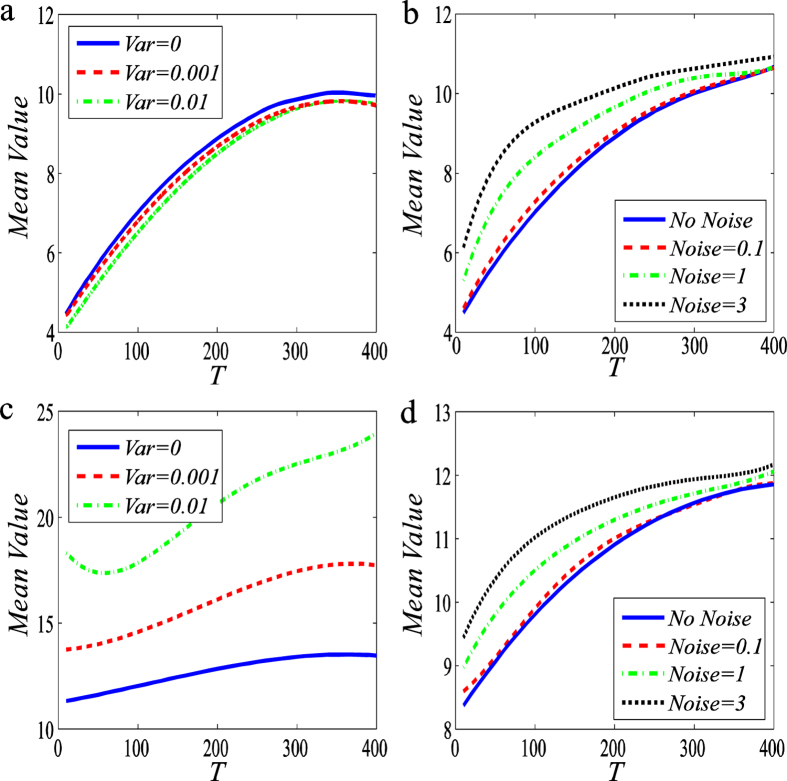
A noisy input signal can induce stochastic focusing in the output mRNA, where *T* represents the length of the whole period of the signal. (**a**,**c**) An amplitude-modulated signal regulates the transcriptioanl rate and the decay rate, respectively. (**b**,**d**) A frequency- modulated signal regulates the transcriptional rate and the decay rate, respectively. In (**a**,**c**), the variance of the input signal is set as 0, 0.001 and 0.01 for the solid blue line, dash red line and dot dash green line, respectively; and the ratio of ‘ON-time’ over ‘OFF-time’ is fixed at 0.25 as *T* is changed. The mean value for the upstream branch of the oscillating amplitude signal is set as 0.8 and the downstream branch as 0.1. The decay rate is 0.05. The value of ‘Duration’ in amplitude-modulated signal is set as 3. In (**b**,**d**), the noise intensity for the input signal is set as 0, 0.1, 1 and 3 for the solid blue, dash red, dot dash green and dot black lines, respectively. In computation, the ratio of ‘ON-time’ over ‘OFF-time’ is fixed at 1 as *T* is varied. The upper bound of the oscillating signal is set as 0.1 whereas the lower bound as 0.025, and the transcriptional rate is fixed at 0.5. Each curve represents the average over 1000 realizations.

As pointed out in the introudction, stochastic focusing (SF) has important biological implications. Different from previously studied cases^16^,^17^,^18^, here we will show that an amplitude-modulated or a frequency-modulated input signal can induce the SF of the mRNA output. See numerical results shown in Fig. 7.

From this figure, we observe that the amplitude-modulted signal can not induce the SF phenomenon in the transcription rate-regulated case, even if the noise intensity increases to a large value (Fig. 7a). Actually, the mean value of the gene product gets only a slightly decreasing in this case. On contrary, in the case that the degradation rate is regulated, the mean mRNA level gets a significant raise with increasing the noise intensity (Fig. 7c). In contrast to the amplitude-modulated signal, the frequency-modulated signal can always induce the shift from a lower level to a higher level of the mean mRNA, independent of the regulatory way. In addition, just like the case of the frequency-modulated signal where the noise can induce the enhancement of information transmission (Fig. 5a and b), the noise also can induce the apparent increase of the mean mRNA level after its intenisty exceeds a certain value (Fig. 7b and d). Except for Fig. 7c, the SF phenomenon is apparent only for the case that the total length of the signal period is below a certain threshold.

SF has important biological implications. Paulsson, *et al*.^16^ ever pointed out that ‘Internal regulation of biochemical reactions is essential for cell growth and survival. Initiation of replication, gene expression, and metabolic activity must be controlled to coordinate the cell cycle, supervise cellular development, respond to changes in the environment, or correct random internal fluctuations. All of these tasks are orchestrated by molecular signals whose concentrations may affect reaction rates of regulated processes.’ In addition, reactive species molecules present often in low copy numbers, so stochasticity of a biochemical system is inevitable. Nature presents multiple intriguing examples of processes that proceed with high precision and regularity. This remarkable stability is frequently counter to modellers’ experience with the inherent stochasticity of chemical reactions in the regime of low-copy numbers. Moreover, the joint effects of noise and nonlinearity can lead to ‘counterintuitive’ behaviour, e.g., SF as demonstrated in a basic enzymatic reaction scheme^18^. Under the assumption of rapid signal fluctuations, SF has been shown the ability to convert a graded response into a threshold mechanism, thus attenuating the detrimental effects of signal noise. The basic premise for generating SF^16^,^18^ is that fluctuations in the ‘input’ species must be sufficiently rapid, so that any rates depending on signalling molecules are of minimal time-correlations.

Unlike the classical enzymatic reaction scheme that can display SF, here we have shown that an ocsillating signal with frequency noise can also induce SF no matter what types of regulation are. In particular, an external signal can induce the SF phenomenon without a rapidly fluctuated rate when compared with the decay rate of gene product (in fact mRNA) in our model. It is worth noting that the noise intensity is an important factor in amplifying the effect of SF. In a word, our result extends the connotation of SF.

## Conclusion and Discussion

Cells survive in complex environments. In order to decode information from their environment, cells often use ligand-bound receptors to trigger the corresponding chemical signals. This in turn intermittently activates TFs that then regulate target genes and their expressions. In such a manner, information is transmitted but energy consumption is accompanied. The conventional view considers that the level of signaling within a cell directly encodes external stimuli, with consequent gradual changes in the nuclear TF concentrations. This is effectively an AM mechanism^4^,^5^,^8^,^38^. However, recent single-cell experiments also showed pulsating signals^1^,^2^,^3^,^38^ and bursty entry of TFs into the cellular nucleus^1^,^33^,^38^, very analogous to FM. In addition, the time-dependent modulation of the transcription or degradation rate may arise from propagation of changes in upstream signals as in fluctuations in regulatory networks^8^,^10^ or it may be a result of intrinsic switching of the gene between ON and OFF states in the absence of any genetic regulation or external signal^39^. In spite of these general descriptions, but how extracellular signals quantitatively and qualitatively affect the intracellular chemical signals is not completely clear. Here, by introducing and analyzing a conceptual model in which the environmental fluctuations are encoded in a transcriptional or degradation rate which varies in time and stochastically, we have found that the regulated gene expression level depends on signal-decoding schemes and characteristics of extracellular signals. Specifically, extracellular noise can induce SF in the FM scheme but only in the AM scheme where an input signal is supposed to regulate the degradation rate of the mRNA. In addition, if the extracellular noise strength remains constant, then enlarging the signal duration can increase the information transmission but decreases the EC; In contrast, if the signal duration remains constant, the greater the information is transmitted, the larger the energy is consumed, meaning that the information transmission is at the cost of energy. Meanwhile, there is an optimal OFF-time such that the information transmission is largest, implying the superiority of asymmetric gene switching. Under the same conditions, the extracellular noise can increase the information transmission if the transcriptional rate is fluctuated, but the effect of this noise is different if the degradation rate is fluctuated. Our findings not only show biological functions of extracellular noise but also verify advantages of the FM scheme in the genetic information processing.

It should be pointed out that the balance between intrinsic noise (e.g., the noise generated in the birth-death process of mRNA in our case) and extrinsic noise (e.g., the noise in the input signal in our case) also plays an important part to condition the amount of information transmission. Cells that implement regulatory circuits or live in rich environments would shift this balance towards intrinsic noise^40^. Beyond this genetic or environmental tuning, cellular systems could avoid the loss of information, due to extrinsic noise, when the input signal operates dynamically rather than statically^41^. Our investigation focused on the effect of an upstream dynamic and noisy signal on the downstream mRNA expression level, but it can also be extended to more complex cases and one could thus interpret the action of several parallel signaling pathways, each conveying approximately one bit of information, as heterogeneous copies of an effective threshold device that enhances information transmission, e.g., this was observed in signaling pathways for the growth factor-mediated gene expression^42^.

Although we have shown that there is a positive correlation between EC and MI (referring to Fig. 4), the related topic is still worth further discussions. R. Landauer ever argued that the erasure of information, which is logically irreversible, is a dissipative process^43^. A direct consequence of this logically irreversible transformation is that the entropy of the environment increases by a finite amount. Despite its fundamental importance for information theory, the erasure principle has not been verified experimentally so far, with the main obstacle being the difficulty of doing single-particle experiments in the low-dissipation regime. Using a system of a single colloidal particle trapped in a modulated double-well potential, Eric *et al*.^44^, established that the mean dissipated heat saturates at the Landauer bound (necessarily produced when a classical bit of information is deleted) in the limit of long erasure cycles. This result, which demonstrates the intimate link between information theory and thermodynamics, highlights the ultimate physical limit of irreversible computation.

As is well known, EC is a global conception and hence an integrative measurement of a system’s behavior. The complexity of information decoding in gene regulation is that the amplitude, frequency and duration of extracellular signals may be dynamic and uncertain (or noisy), and may affect both the EC and the information transmission (quantified by MI). From a viewpoint of biology, gene regulatory networks may follow some design principles for optimal evolutionary fitness, implying that gene expression is locally and globally constrained^45^,^46^,^47^,^48^,^49^. One constraint is on information transmission^45^,^46^,^47^. We have shown that the external noise can increase the information transmission, implying that the extracellular noise would be beneficial for genetic regulation, namely the regulated gene may use this noise to achieve its function. Meanwhile, the gene transducing network is expected to minimize the EC^46^,^48^,^49^. We have seen that in the case that the timescale of an extracellular signal becomes slow and the noisy environment is kept the same, the EC may decrease while the information transmission may increase, indicating that a slow process or slow switching is superior to other cases. These results are consistent with the expression modulation of eukaryotic cells^37^. And the external noise may induce SF in the FM scheme, but this phenomenon takes place only in the AM scheme with degradation modulation, indicating that the former scheme is more efficiency than the latter scheme in the same cellular environment. Thus, when the “information criterion” and “minimum energy criterion” as well as “efficiency criterion” are considered simultaneously, there would be a trade-off relationship between them, which is worth further investigation.

Broadly speaking, temporal ordering (regularity and periodicity) serves at least two roles in a living system: energy extraction from the environment and information processing^41^,^50^,^51^. We believe that further work should combine single-cell experiments with the ideas of collective behavior and engineering principles.

## Additional Information

**How to cite this article**: Liu, P. *et al*. The dynamic mechanism of noisy signal decoding in gene regulation. *Sci. Rep.* **7**, 42128; doi: 10.1038/srep42128 (2017).

**Publisher's note:** Springer Nature remains neutral with regard to jurisdictional claims in published maps and institutional affiliations.

## Supplementary Material

Supplementary Information
