# Supplementary material for: The dynamic mechanism of noisy signal decoding in gene regulation
Source: Sci Rep. 2017 Feb 8;7:42128. doi: 10.1038/srep42128 (PMC5296728; doi:10.1038/srep42128)
Supplement: Supplementary Information [file srep42128-s1.pdf]

Supplementary information for

The dynamic mechanism of noisy signal decoding in gene regulation

Peijiang Liu, Haohua Wang, Lifang Huang and Tianshou Zhou

Appendix A. A modified version of the Gillespie algorithm

The standard Gillespie algorithm[1] can be used to simulate a chemical system where the reaction propensities are time-independent. Since the transcription rate or degradation rate is dynamics or stochastic in our case, we need a modified version of this algorithm to account for the rate-fluctuating gene expression model. In the case that the transcriptional rate is random (similar for the degradation rate), this modified algorithm consists of the following main steps.

Step-1 Input initial conditions (including the final time t_{final} and the initial time t that is set as zero) and parameter values.

Step-2 Generate a noisy oscillating signal.

A: For a FM signal, ON-Time and OFF-Time are sampled from a log-norm distribution, the mean and the variance of which are pre-given;

B: For an AM signal, ON-Time and OFF-Time are set as constants but the fluctuation in amplitude is sampled from a log-norm distribution with pre-given mean and variance;

C: the signal duration is sampled from one point distribution.

Step-3 Calculate the propensity function for each chemical reaction.

Case A: For a FM signal, let t_n be the time for the next discontinuous change in $k_b(t)$ or $k_d(t)$ in the reaction. $k_b(t)$ or $k_d(t)$ takes the value of the left-side time t_n if $t < t_n$, and the value of the first right-side time t_n if $t > t_n$.

Case B: For an AM signal, the following two indices should be taken into consideration: the one is the time for the signal switching from the upper branch to the

lower one, which is set as t_n, and the other is the times between two successive pulses of the signal, which are set as t_f and t_s. Note that no matter how $t < t_n$ or $t > t_n$, there must be two values, denoted by t_f and t_s, such that they satisfy the condition $t_f < t < t_s$. Thus, we determine the value of $k_b(t)$ or $k_d(t)$ at the time point t_f. Based on the reaction rates determined in such a manner, we calculate the propensity function for each chemical reaction.

Step-4 For each reaction, denoted by μ, we generate a putative next reaction time, denoted by τ_μ.

Step-5 Let t_n be the time for signal switching from the upper branch to the lower one. If $t + \tau_\mu < t_n$, then we change the number of mRNAs appropriately for the occurrence of reaction μ, and change t to $t + \tau_\mu$. If $t + \tau_\mu > t_n$ then we change $k_b(t)$ or $k_d(t)$ accordingly, and set $t = t_n$.

Step-6 If $t > t_{final}$, then exit (or end). Otherwise, go to step-3.

Note that if the variance of the random variable following the lognormal distribution is equal to zero, the external signal corresponds to the deterministic one. In order to simulate an arbitrarily given function or a known stochastic process, we first need to approximate this function by a series of step functions or by a piece-wise linear function and then proceed with the time interval of duration as set above.

Appendix B. Mutual information

Information theory quantifies information transduction across a channel between a signal and a response. For two discrete random variables X and Y, representing the signal input and response output respectively, the mutual information (MI)[2,3], measured in bits, quantifies the reduction in uncertainty about the response output Y given the knowledge of the signal input X, and is defined as:

$$MI(X;Y)=\sum_{y\in Y}\sum_{x\in X}p(x,y)\log_2\frac{p(x,y)}{p(x)p(y)} \tag{A.1}$$

where $p(x,y)$ is the joint probability distribution function of X and Y; the response distribution, $p(y)$, is the measured distribution of the output product of gene expression; the signal distribution, $p(x)$, is the relative probability of extracellular signal which may be fluctuated in amplitude, frequency or duration, respectively.

Alternatively, MI of signal input X and response output Y can be expressed as:

$$MI(X;Y)=H(Y)-H(Y\,|\,X) \tag{A.2}$$

where $H(Y)=-\sum_{y\in Y}p(y)\log_2 p(y)$ is the marginal entropy of response output Y and $H(Y\,|\,X)$ is the entropy of Y conditional on signal input X (called the conditional entropy). In order to calculate MI, we generate the MI of two time-series, the signal input $k_b(t)$ (or $k_d(t)$) and the response output $m(t)$, where $k_b(t)$ (or $k_d(t)$) and $m(t)$ are the transcriptional rate (or degradation rate) and the mRNA level at time t, respectively.

Appendix C. Energy consumption

Generally, if a system exchanges signals with its surroundings, i.e., if a system is open, then this system usually approaches a steady state that however is not an equilibrium. The most distinguished characteristics of non-equilibrium steady-state (NESS) is that the underlying system has a nonzero flux or a nonzero chemical potential gradient. That is, the system needs energy consumption to sustain its steady-state[4,5]. For an irreversible Markov process with the state labeled by σ and the transition probability from σ to σ' denoted by $k(\sigma,\sigma')$, if we denote by $P(\sigma)$ the steady-state probability in state σ, then the entropy production rate (denoted by EP) for an NESS is calculated according to the following general formula[5-7]:

$$EP = \sum_{\sigma,\sigma'} P(\sigma)k(\sigma,\sigma')\log\frac{k(\sigma,\sigma')}{k(\sigma',\sigma)} \tag{A.3}$$

It is not difficult to show that in our case, this formula becomes

$$EP(t) = \sum_{m} P(m,t)\left[k_b(t)\log\frac{k_b(t)}{k_d(t)(m+1)} + k_d(t)m\log\frac{k_d(t)m}{k_b(t)}\right] \tag{A.4}$$

where $EP(t)$ is the entropy production rate for the open system at time t; $P(m,t)$ represents the probability that the gene generates m mRNA molecules at time t; $k_b(t)$ and $k_d(t)$ are the transcription and degradation rates, respectively. According to the main text, we know

$$P(m,t) = \frac{1}{m!}e^{-\mu(t)}\mu^m(t), \text{ where } \mu(t) = e^{-\int_0^t k_d(x)dx}\int_0^t k_b(y)e^{\int_0^y k_d(x)dx}dy \tag{A.5}$$

Thus, substituting Eq. (A.5) into Eq. (A.4) yields

$$EP(t) = \left[k_b(t) - k_d(t)\mu(t)\right]\left[\log\frac{k_b(t)}{k_d(t)} - e^{-\mu(t)}\sum_m \frac{\mu^m(t)}{m!}\log(m+1)\right] \tag{A.6}$$

Essentially, formula (A.4) or (A.6) describes how individual reaction rates quantitatively affect energy consumption. However, obtaining qualitative results directly from this formula seems difficult and needs to resort to numerical calculation.

References

1. Berg, O. G., Paulsson, J. & Ehrenberg, M. Fluctuations in repressor control: thermodynamic constraints on stochastic focusing. Biophys. J. **79**, 2944-2953 (2000).

2. Hansen, A. S. & O'Shea, E. K. Limits on information transduction through amplitude and frequency regulation of transcription factor activity. Elife **4,** e06559 (2015).

3. Tanase-Nicola, S., Warren, P. B. & Ten Wolde, P. R. Signal Detection, Modularity, and the Correlation between Extrinsic and Intrinsic Noise in Biochemical Networks. Phys. Rev. Lett. **97**, 068102 (2006).

4. Marquez-Lago, T. T. & Stelling, J. Counter-intuitive stochastic behavior of simple gene circuits with negative feedback. Biophys. J. **98**, 1742-1750 (2010).

5. Ochab-Marcinek, A. &Tabaka, M. Bimodal gene expression in noncooperative regulatory systems. Proc. Natl. Acad. Sci. USA. **107**, 22096–22101 (2010).

6. Wang, H. H, Yuan, Z. J., Liu, P. J. & Zhou, T. S. Mechanisms of information decoding in a cascade system of gene expression. Phys. Rev. E **93**, 052411 (2016).

7. Nandi, S. & Ghosh, A. Transcriptional dynamics with time-dependent reaction rates. Phys. Biol. **12,** 016015 (2015).
